# Optimizing drug–target interaction prediction based on random walk on heterogeneous networks

**DOI:** 10.1186/s13321-015-0089-z

**Published:** 2015-08-19

**Authors:** Abhik Seal, Yong-Yeol Ahn, David J Wild

**Affiliations:** Indiana University Bloomington, School of Informatics and computing, Bloomington, USA

**Keywords:** Random walk with restart, Prediction, Drug, Targets, Chemical fingerprints

## Abstract

**Background:**

Predicting novel drug–target associations is important not only for developing new drugs, but also for furthering biological knowledge by understanding how drugs work and their modes of action. As more data about drugs, targets, and their interactions becomes available, computational approaches have become an indispensible part of drug target association discovery. In this paper we apply random walk with restart (RWR) method to a heterogeneous network of drugs and targets compiled from DrugBank database and investigate the performance of the method under parameter variation and choice of chemical fingerprint methods.

**Results:**

We show that choice of chemical fingerprint does not affect the performance of the method when the parameters are tuned to optimal values. We use a subset of the ChEMBL15 dataset that contains 2,763 associations between 544 drugs and 467 target proteins to evaluate our method, and we extracted datasets of bioactivity ≤1 and ≤10 μM activity cutoff. For 1 μM bioactivity cutoff, we find that our method can correctly predict nearly 47, 55, 60% of the given drug–target interactions in the test dataset having more than 0, 1, 2 drug target relations for ChEMBL 1 μM dataset in top 50 rank positions. For 10 μM bioactivity cutoff, we find that our method can correctly predict nearly 32.4, 34.8, 35.3% of the given drug–target interactions in the test dataset having more than 0, 1, 2 drug target relations for ChEMBL 1 μM dataset in top 50 rank positions. We further examine the associations between 110 popular top selling drugs in 2012 and 3,519 targets and find the top ten targets for each drug.

**Conclusions:**

We demonstrate the effectiveness and promise of the approach—RWR on heterogeneous networks using chemical features—for identifying novel drug target interactions and investigate the performance.

**Electronic supplementary material:**

The online version of this article (doi:10.1186/s13321-015-0089-z) contains supplementary material, which is available to authorized users.

## Background

Recent work has demonstrated the power of network-based approaches in drug discovery [1–3]. We have shown previously that a large semantic network of drug–target interactions provides a powerful framework for predicting new associations [4] and that an algorithm that predict drug-target associations by using this network performs surprisingly well, even without training datasets or incorporating target preference [5].

In this work, we apply a random walk-based link prediction algorithm based on Chen et al. [3] to a more extensive drug–target network and evaluated its performance using an external dataset. We combine three networks—drug–drug, target–target, and drug–target—to construct a heterogeneous network of drugs and targets. The links between drugs are obtained by quantifying molecular similarity with chemical fingerprints and examining the shared targets. The links between targets are obtained by calculating sequence similarity between proteins and again examining the shared drugs. Finally, the links between drugs and targets are obtained from DrugBank [6].

Random walk is a useful mathematical framework that provides a systematic way to measure importance of nodes in a network. The most widely known is the PageRank algorithm [7]. PageRank, developed for ranking web pages, measures page clicks of hypothetical web surfers who randomly click hyperlinks in the network of webpages. Since it is possible for the surfer to be trapped in a dead-end webpage that does not have any outgoing link, at each time step the surfer may jump to a random webpage with a probability *c*. Interestingly, this formulation also provides a simple way to define a random walk-based “distance” from a node *a* (or a set of nodes) to every other node, namely by allowing the random walkers to jump only to the source node *a* (or the source set of nodes) and restart from there. As a result, it is more likely to find the random walker at the vicinity of the source node than at a distant part of the network, and thus we are able to estimate the relevance (closeness) of each node with respect to the source node. The prediction method applies this idea to identify drugs and targets that are relevant to a set given set of drugs and targets.

Consider an undirected, unweighted network *G* = (*V*, *E*), where *V* is the set of nodes and *E* is the set of links. For each pair of nodes $a, b \in V$ we can assign a proximity score by executing the following procedure: (1) we start a random walker from *a.* (2) At each time step, with the probability 1 − *c*, the walker walks to one of the neighbors, *b*, according to the transition probability matrix *W*_*ab*_ = *S*_*ab*_/*K*_*a*_, where *S*_*ab*_ is the adjacency matrix of the network and (*S*_*ab*_ equals 1 if node *a* and *b* are connected, 0 otherwise) *K*_*a*_ denotes the degree of *a*. (3) With the probability *c*, the walker goes back to *a*. (4) After many time steps the probability of finding the random walker at node *x* converges to the steady-state probability, which is our proximity score *S*_*a*→*x*_. This approach allows us to generate candidate targets for a given drug even if the drug does not have any known targets. If there is a missing interaction between drug *d* and target *t*, we expect that *d* is more likely to interact with other targets that are similar to *t*, and drugs that are similar to *d* are more likely to interact with *t*. Therefore, we expect to see many indirect paths that connect *d* and *t*. These indirect paths are likely to be captured by the random walkers.

## Methods

We apply the RWR algorithm to a drug–target network and use an external dataset extracted from ChEMBL 15 (544 drugs and 467 proteins) at bioactivity cutoff points of 10 and 1 μM to quantitatively evaluate the performance and robustness of the approach.

### Datasets

#### Drugs

We compile a set of approved drugs from DrugBank database (Version 3.0) [6], consisting of 727 compounds and 3519 protein targets (Additional file 1). To construct the network between drugs, we incorporate two types of similarity measures: chemical (structural) similarity and target similarity. We calculate chemical similarity between drugs by using the Jaccard Index (Tanimoto Coefficient) between their chemical fingerprints. The Jaccard Index is defined as the size of the intersection of two sets divided by the size of the union of the sets, ranging between 0 and 1. For binary vectors like chemical fingerprints, it is defined as *C*/(*A* + *B* − *C*) where *C* is the number of bits in common, *A* is the number of bits in one of the fingerprints, and *B* is the number of bits in the other fingerprint. We use four types of chemical features namely, MDL MACCS166 keys (fragmental descriptors) [8], ECFP6 fingerprints (extended connectivity fingerprint path 6) [9], 2D Pharmacophore fingerprints (PHFP4) [10] and ROCS program which uses Tanimoto combo similarity—which combines shape and color measures of a compound, we calculate them with ROCS program [11].

ECFP (extended connectivity fingerprint) encodes information on atom-centered fragments that is derived from the variant of the Morgan algorithm [12]. ECFPs are generated using the neighborhood of each non-hydrogen atom into multiple circular layers up to a given diameter. These atom-centric substructural features are then mapped into integer codes using a hashing procedure, which constitute the extended-connectivity fingerprint. ECFP can, for instance, represent a very large number of features (over 4 billion), do not rely on predefined dictionary of features, can represent stereochemical information, and can be interpreted as the presence of particular substructures. 2D pharmacophore fingerprints are calculated using topological (bond) distances.

**Fig. 1:**
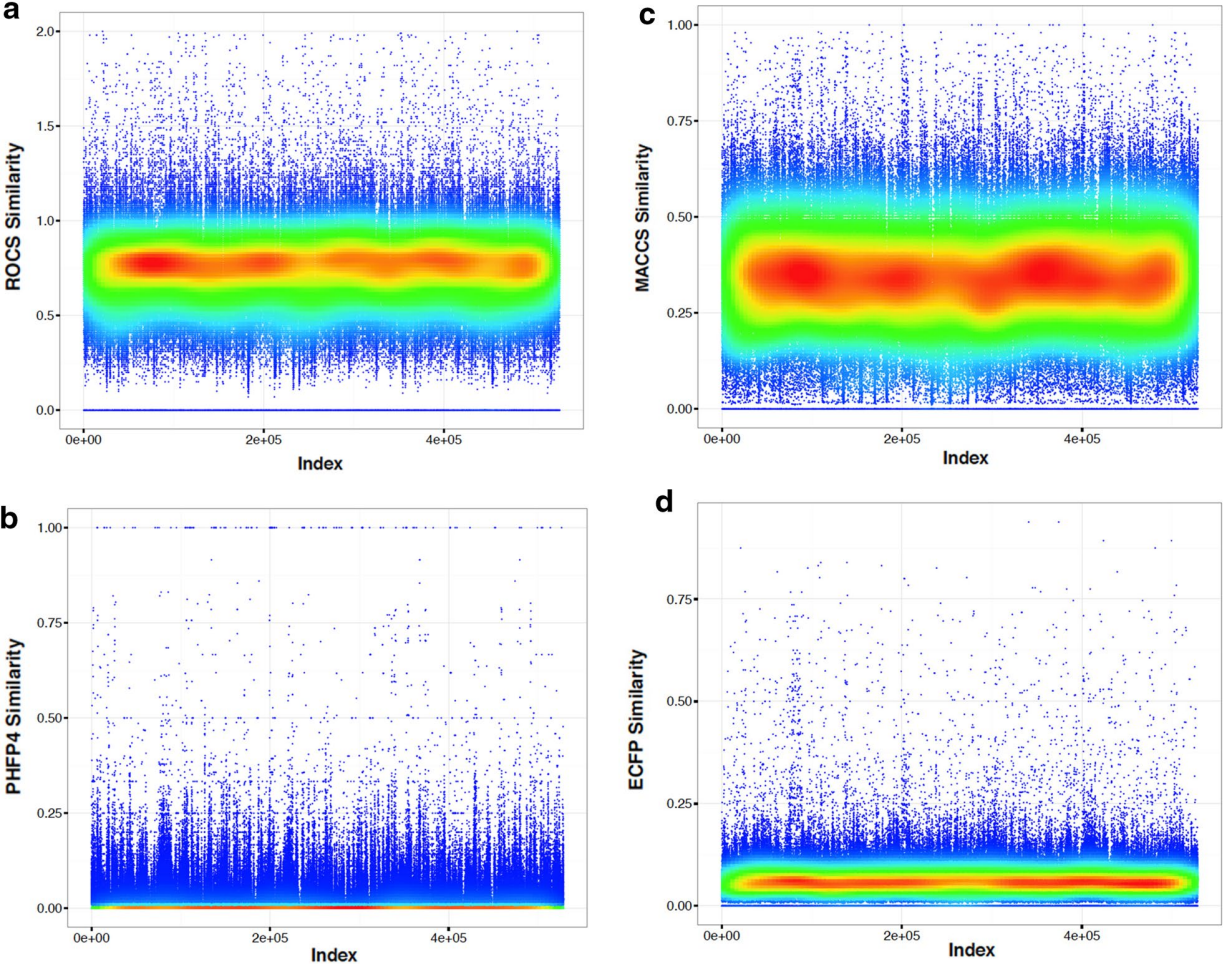
Plots showing the compounds similarity distributions according to the four types of Chemical fingerprints. **a** ROCS similarity, **b** PHFP4 similarity, **c** MACCS similarity, **d** ECFP similarity.

Pharmacophore fingerprints consist of pairs, triplets, or quartets of molecular features and the corresponding bond distances among them. We use PHFP_4 (quartets which includes number of bonds in the shortest path between the features) fingerprints for the calculation. The feature vectors of quartets involve four pharmacophoric features, six Euclidean distances separating those features, and an indication of chirality. For 3D alignment and similarity we used ROCS 3.2, which is a shape-similarity method based on the Tanimoto-like overlap of volumes. The alignment was developed using the Combo score, which combines the Tanimoto shape score with the color score that added the score for the appropriate overlap of groups with similar properties (donor, acceptor, hydrophobe, cation, anion, and ring) [http://docs.eyesopen.com/rocs/shape_theory.html] defined by SMARTS. Conformers for the data set is created using OMEGA [13], about 250 conformers with RMSD threshold of 0.6 is generated. ROCS performs shape-based overlay of conformers as atom-centered Gaussian functions. ROCS score performed in color optimization mode where it optimizes the molecular overlay to maximize both the shape overlap and the color overlap obtained by aligning groups with the same properties that are contained in the color force field file. This overlay is then subsequently scored using the sum of shape Tanimoto for the overlay and the color score called Tanimoto combo score. We use *C*_*s*_ to refer the *N* by *N* chemical compounds similarity matrix. For the 727 drugs we used different chemical descriptors to calculate the Tanimoto similarity distribution to create a view of how similar the drugs look like. The distributions of different similarities Fig. 1 shows that for four fingerprints (166 MACCS Keys, PHFP4, 3D ROCS, and ECFP6), 0.56% had a similarity above 0.7 for the MACCS keys, 0.31% had similarity above 0.4 for PHFP4, 0.88% had similarity above 1.2 Tanimoto Combo score for ROCS, 0.24% had similarity above 0.3 for ECFP6. The mean similarity is 0.346, 0.019, 0.742, and 0.063 for MACCS, PHFP4, ROCS, ECFP6 fingerprints, respectively. This indicates how diverse chemical structures are in the drug dataset (Additional file 2).

#### Proteins

We extracted 3,519 target proteins across all available species and their sequences from the DrugBank database. As proteins in other species may provide useful information in our network-based approach, we keep all the proteins regardless of species. Note that, human proteins still dominate the dataset. We calculate the sequence similarity matrix *T*_*s*_ by using the R Biostrings package and the normalization procedure proposed by Bleakley and Yamanishi [2]:

1 $$ T_{s} = \frac{{SW\left( {g,g^{'} } \right)}}{{\sqrt {SW\left( {g,g} \right)} \sqrt {SW\left( {g^{'} ,g^{'} } \right)} }}, $$

where SW (∙, ∙) means the original Smith–Waterman similarity score.

#### Drug target matrix

**Fig. 2:**
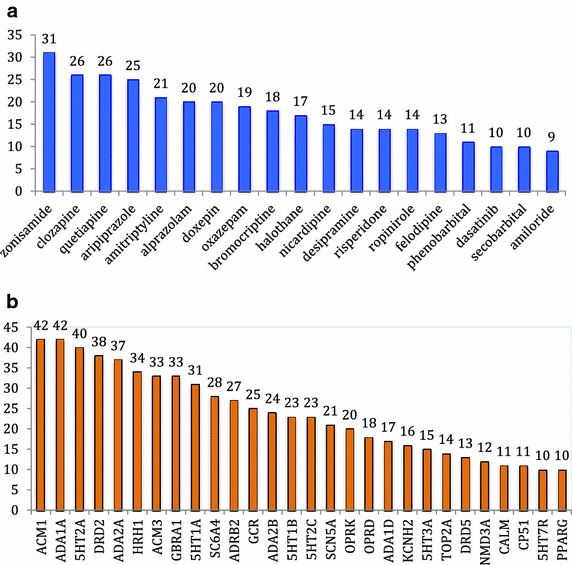
**a** drugs with the most target associations and **b** targets with the most drug associations.

We construct a drug-target relationship matrix *A* whose element *A*(*i*,*j*) is 1 if drug *i* interacts with target *j*, otherwise 0. The matrix is sparse; the total number of connections among the drugs and targets is only 2,557, with 687 drugs having at least one known target and with 628 proteins having at least one drug. There are 73 connected components in the whole drug target network dataset. The largest connected component in this bipartite graph has 498 drugs and 279 proteins. The connections are concentrated to a small number of drugs (see Fig. 2a) that affect nervous systems mostly psychoanaleptics and psycholeptics have the largest number of interactions. As most drugs are metabolized by cytochrome p450, which serves as an important protein target and enzyme for the drugs, the interaction between important enzymes CYP3A4, CYP2D6 and CYP3A5 are not considered on the drug target interaction matrix except for the drug paliperidone, which has interactions to all the three cytochromes targets mentioned above.

Figure 2b exhibits the targets that interact with most number of drugs. The top frequent targets are Muscarinic receptor (ACM1), Adrenoreceptor alpha 1A (ADA1A), Histamine receptors (5HT2A), and dopamine receptors (DRD2). In addition to the drug–drug similarity matrix *C*_*s*_ (based on chemical similarity) and target–target similarity matrix *T*_*s*_ (based on sequence similarity), we introduce additional measure of drug–drug and target–target similarities based on the network structure. $C_{s}^{n}$ is a drug–drug similarity matrix based on the number of shared targets between drugs; $T_{s}^{n}$ is a target–target similarity matrix based on the shared drugs. The similarity between two drugs *d*_*i*_ and *d*_*j*_ is quantified by Jaccard coefficient, which is defined by:

2 $$ C_{s}^{n} (d_{i} ,d_{j} ) = \frac{{M_{l} (i,j)}}{{M_{l} \left( {i,i} \right) + M_{l} \left( {j,j} \right) - M_{l} (i,j)}}, $$

where *M*_*l*_ is the inner product of the drug–target interaction matrix. The similarity between targets is defined in the same manner.

We define the final drug–drug similarity matrix *S*_*d*_ by taking a linear combination of the chemical similarity matrix (*C*_*s*_) and target sharing similarity matrix ($C_{s}^{n}$). Similarly, the final target–target similarity matrix *S*_*t*_ is calculated using the sequence similarity matrix (*T*_*s*_) and drug sharing similarity matrix $(T_{s}^{n} )$.

3 $$ {S_{d} = \, w_{d} C_{s} + \, \left( {1 - w_{d} } \right)C_{s}^{n} } $$

4 $$ S_{t} = \, w_{t} T_{s} + \, \left( {1 - w_{t} } \right)T_{s}^{n} $$

### Random walk with restart implementation

We combined drug–drug, drug–target, and target–target networks into a undirected heterogeneous network. Many nodes have connections to both drugs and targets and we call them *bridge nodes*. At a bridge node, a random walker may jump to a node with the other type or to a node with the same type. The probability to do so is *λ* and *1* – *λ*, respectively. For instance, if a random walker is at a drug node, it can jump to one of the connected target nodes with the probability *λ*, or jump to connected drug nodes with the probability *1* − *λ*. We call the parameter *λ* the *jumping probability*. If *λ* is 0, a random walker will explore only one type of networks. Most importantly, the probability *p*_∞_(*i*) is the probability of finding the random walker at node *i* in the steady state. It gives a measure of probability of source and target node (proximity) between node *i* and the source nodes where the random walks restarts.

The transition matrix is represented by,

$$ W = \left[ {\begin{array}{*{20}c} {W_{TT} } & {W_{TD} } \\ {W_{TD} } & {W_{DD} } \\ \end{array} } \right] $$

Here *W*_*TT*_ is the target to target transition matrix, *W*_*DD*_ is the drug to drug transition matrix, *W*_*DT*_ is drug to target transition matrix and *W*_*TD*_ is target to drug transition matrix. The calculation of each of the transition matrix in discussed in Chen et al. [3]. The random walk is implemented on the heterogeneous network using the Eq. (5) given below

5 $$ p_{t + 1} = \, \left( {1 - c} \right)W^{T} p_{t} + \, cp_{0} $$

*p*_*t*_ is a vector in which *i*th elements holds the probability of finding the random walker at node *i* at time step *t*. Initial probability vector ***p***_0_ controls the restart probability *c*.

6 $$ p_{0} = \left[ {\begin{array}{*{20}c} {(1 - \eta )u_{0} } \\ {\eta v_{0} } \\ \end{array} } \right]\left( {\text{initial probability matrix}} \right) $$

*u*_0_ and *v*_0_ be the initial probability vectors for target network and drug network, respectively. Parameter ƞ controls the importance of two kinds of seed nodes, i.e. drug node and target node. We tested the importance parameter $\eta$ for different values ranging from 0 to 1.

After a number of iteration steps, the *p*_*t*_ converges to a steady-state probability vector *p*_*∞*_, where $$ p_{\infty } = \left[ {\begin{array}{*{20}c} {u_{\infty } } \\ {v_{\infty } } \\ \end{array} } \right] $$. In practice, we consider *p*_*t*_ = *p*_∞_ if the change between *p*_*t*_ and *p*_*t* + *1*_ (measure by the Frobenius norm) is less than 10^−10^.

For finding novel targets for a given drug, we set the drug and the targets that are directly connected to the drug as our seed nodes. Suppose that there are six targets *T*_*1*_,…,*T*_*6*_ and four drugs *D*_*1*_, *D*_*2*_, *D*_*3*_, and *D*_*4*_. We focus on drug *D*_*3*_ and tries to find novel targets for D3. We already know that *D*_*3*_ interacts with *T*_*2*_ and *T*_*3*_. Then *T*_1_, *T*_4_, and *T*_5_ are candidate targets for drug *D*_3_. We set *T*_2_, *T*_3_, and *D*_*3*_ as the source nodes, namely *u*_*0*_ = [0,1,1,0,0,0]^*T*^ and *v*_*0*_ = [0,0,1,0]^*T*^

The stationary probability *p*_∞_ represents the expected relevance of each drugs and targets regarding the source node set *T2*, *T3* and *D3*. For instance, if the value for *T1* is the largest among *T1*, *T4* and *T5*, then we expect that *T1* is most likely to interact with *D3*.

## Results and discussion

### Evaluating target prediction performance using link perturbation

The network-based method aims to predict new targets for a given drug. We evaluated our approach using a perturbed network where we have removed some links to measure how well our approach re-identifies those removed links. There are five parameter to explore: the restart probability *c*, the jumping probability *λ*, the relative importance ƞ, which controls the relative importance between two types of seeds, *w*_*d*_ and *w*_*t*_ that weigh the drug and target similarity matrices and network based similarity measure of the drugs and proteins, respectively. Among these five parameters, we have tested ƞ because, to our knowledge, the restart probability *c*, jumping probability *λ*, and *w*_*d*_ and *w*_*t*_ are not likely to affect the results in a significant way. First, it is known that in most cases the choice of restart probability *c* does not affect performance of PageRank algorithm and other PageRank-based algorithms. For instance, the results of PageRank are highly insensitive to the choice of restart probability [14, 15]. It has been shown that the prediction results from RWR are also robust [7, 14–16]. Because of these evidences, we here simply adopt the previously used value of 0.3 [3]. Second, the robustness of *λ* (jumping probability) has already been discussed [15–17]. It has been shown that the weight parameters *w*_*d*_ and *w*_*t*_ are robust among the prediction results [3].

In our drug target network 684 (94%) drugs have at least one target. We prepare a test network of 684 drugs where we remove one links from 684 drugs with a total of 684 drug–target interactions. The links include drugs which has only one target in order to see if the method able to predict single known interaction. We check how many missing links are in top *N* of the ranked list. We divide the number of actual targets that are in the top *N* lists by the number of tests (684) and call the fraction as ‘*recoveredfraction*’. We also used a random set to calculate the statistics with same parameters and found that the results are way better than random set. We tested our results with different values of w_d_ and w_t_ ranging from 0 to 1 and found that at extreme point like 0 and 1 the performances drops radically but the performance gets best on values of w_d_ and w_t_ of 0.5 given in Additional file 3: Sheet 3. We test different values of $\eta$ for the four different chemical fingerprints to identify the optimal value of $\eta$ and the right of chemical features. We observed that the prediction performance becomes optimal when $\eta$ is small but not 0. We found optimal performance at $\eta = 0.0 1$. For all the other values of $\eta \left( {0. 1- 0. 9} \right)$ the prediction rate for all fingerprints is equal. The values are given in the Additional file 3: Sheet 1. We find nearly 28% of the true interactions out of 684 can be retrieved at the top 10 rank positions and more than 38% of the interactions can be retrieved at the top 50 rank positions. We also prepare 10 test networks of drugs that have more than two targets links, where we randomly remove 100–1,000 links. Using the 10 test networks we predicted the removed links. We repeat this process, from preparing a test network to calculating the recovered fraction, 50 times to obtain the ‘*average recovered fraction*’. From Table 1 we can see that if we remove 100 links it gave us the best prediction rates and as we increase the number of removed links to 1,000 the prediction rates falls. From Table 2 shows the recovered fraction rates for top 10, 25, 50, 100, 200, 500, 1,000 retrieved targets we also find almost 32% of the true interactions can be retrieved at the top 10 rank positions for each of the test networks and more than 75% of the true interactions can be retrieved at the top 50 rank positions. This indicates that the method performs well if we remove links from drugs which are having at least two or more known interactions, since it uses the given interaction information in the network. We also measured the area under accumulation curve, area under ROC curve AUC (Top 10%), BEDROC and enrichment factor given in Table 1. The area under the receiver operating characteristic (ROC) curve (AUC) is widely used to evaluate the performance of the ranking method. The advantage of using AUC is, the value ranges from 0 to 1 with 0.5 corresponding to randomness. Another key criterion for measuring the success of ranking prediction is the enrichment of annotated associations among top ranking associations. The higher the percentage of annotated associations among the top ranking associations, the better the performance of the prediction. The enrichment criterion is evaluated by enrichment factor (EF) [16, 17]. EF reflects the capability of a screening application to detect true links (true positives) compared to random selection. Thus, its value should always be greater than 1 and the higher it is, the better the enrichment performance. When we are predicting links it should rank true links in the top-ranking list. Metric likes ROC not sensitive to early recognition for example considering cases like where (1) true links are retrieved at beginning of a rank ordered list, (2) where true links are randomly distributed and (3) where true links, which are retrieved in the middle of the rank, ordered list. In all of the above cases ROC is 0.5 but in terms of early recognition we see that case (1) is better than (2) and (3). To overcome these limitations methods such as RIE and BEDROC have been proposed. By changing the tuning parameter, α, one can test whether the method is able to rank true links early or not.

**Table 1:** Shows the recovered fraction values for top 10, 25, 50, 100, 200, 500 and 1,000 ranks with the number of links removed

Number of links removed	AUAC	AUC	BEDROC	EF	AUC (top 10%)
100	0.947	0.991	0.833	9.23	0.867
200	0.938	0.995	0.827	9.100	0.857
300	0.930	0.995	0.818	8.95	0.845
400	0.920	0.991	0.805	8.79	0.830
500	0.916	0.997	0.801	8.71	0.824
600	0.908	0.995	0.789	8.56	0.812
700	0.899	0.981	0.780	8.42	0.802
800	0.885	0.997	0.761	8.20	0.783
900	0.869	0.955	0.741	7.91	0.765
1,000	0.854	0.956	0.715	7.62	0.741

**Table 2:** Shows the recovered fraction values for top 10, 25, 50, 100, 200, 500 and 1,000 ranks with the number of links removed

# Of links removed	TOP 10 (%)	TOP 25 (%)	TOP 50 (%)	TOP 100 (%)	TOP 200 (%)	TOP 500 (%)	TOP 1,000 (%)
100	32.24	78.24	87.76	90.74	91.92	93.22	93.88
200	31.92	77.95	87.26	89.86	91.15	92.37	93.12
300	32.14	78.31	86.82	89.48	90.68	91.80	92.63
400	32.04	77.4	85.34	88.07	89.24	90.33	91.45
500	32.62	77.39	85.04	87.56	88.70	89.95	91.1
600	32.53	76.21	83.68	86.23	87.54	88.86	90.16
700	32.5	75.64	82.69	85.18	86.57	87.89	89.33
800	33.06	74.13	80.88	83.45	84.86	86.35	87.97
900	33.58	72.14	78.49	81.04	82.77	84.57	86.38
1,000	33.71	69.81	76.008	78.31	80.22	82.12	84.42

We found that the performance of the algorithm for ranking the targets by different chemical features is approximately same which indicates using this approach a user can identify protein targets with any one set of chemical features. We used public 166 MACCS keys, ECFP6, PHFP4 and 3D ROCS to perform the analysis and it is surprising that the commercial programs feature performance is same as the 166 public MACCS keys.

As a baseline, we test how RWR results differ from the results of random set of interactions. We randomized the interactions and similarity matrices and performed RWR and found the random set prediction rate was way below our original prediction rate as given in Additional file 3: Sheet 1.

### Evaluating target prediction performance using an external dataset (ChEMBL)

In addition to the internal evaluation using link perturbation approach, we evaluate the performance of our method using an external dataset, namely ChEMBL version 15 database.

**Fig. 3:**
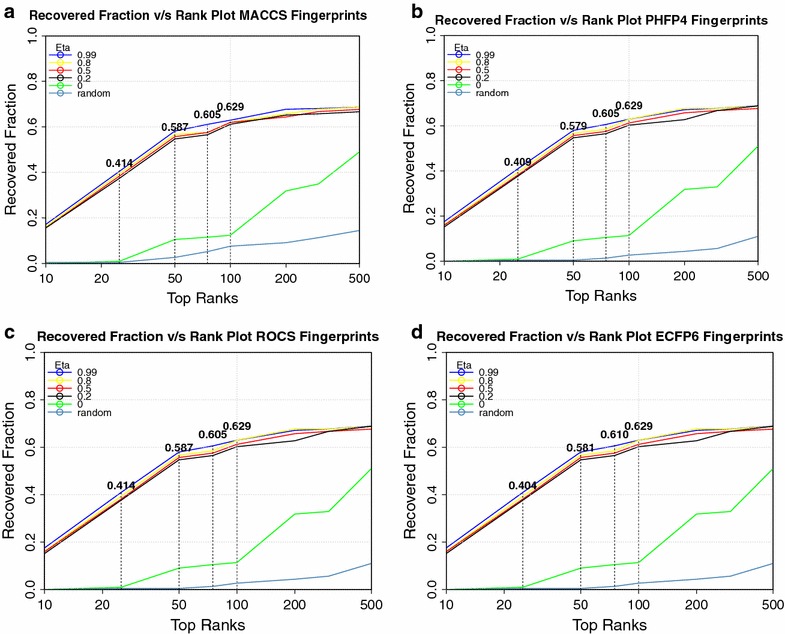
Showing the recovered fractions against the rank with different $\eta$ (eta) values for ChEMBL datat at 1 μM cutoff. The *recovered fraction is* calculated by the number of targets retrieved at different rank positions over total number true interactions. **a** MACCS, **b** PHFP4, **c** ROCS, **d** ECFP4.

**Fig. 4:**
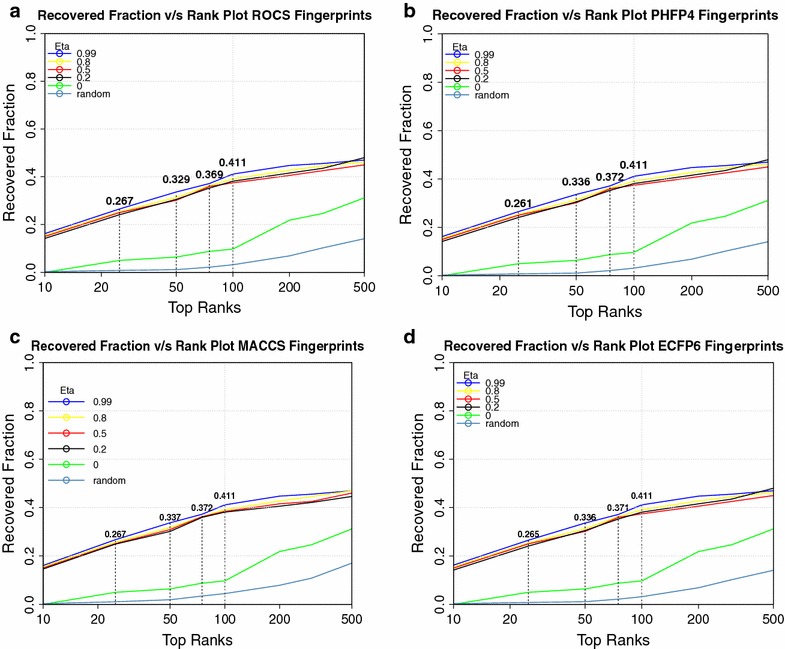
Showing the recovered fractions against the rank with different $\eta$ (eta) values for ChEMBL data at 10 μM cutoff. The *recovered fraction is* calculated by the number of targets retrieved at different rank positions over total number true interactions. **a** MACCS, **b** PHFP4, **c** ROCS, **d** ECFP4.

From ChEMBL 15 data we extract all the drugs and targets that have activity values not more than 1 μM (Additional file 3: Sheet 4) and 10 μM (Additional file 3: Sheet 4) with units IC50, Ki, Kd, EC50, AC50, LC50, and GI50. Our training model is based on DrugBank and UniProt database so we mapped the drugs and targets ChEMBL ids with the DrugBank ids and UniProt ids. We used Pubchem mapping tool (http://pubchem.ncbi.nlm.nih.gov/idexchange/) to map ChEMBL ids to DrugBank ids and the UniProt mapping tool (http://www.uniprot.org/?tab=mapping) to map target ChEMBL ids to UniProt ids. It gives us 544 drugs and 467 protein targets, with 3,463 and 564 drug target interactions those are below 10 and 1 μM, respectively. Naturally, there are lots of interactions that are present in both DrugBank and ChEMBL. We tested performance of parameter $\eta$ at different values on ChEMBL 1 μM set and 10 μM having which have more than 0, 1 and 2 target relations. Figures 3 and 4 shows the recovered fractions against the rank with different $\eta$ (eta) values for ChEMBL datat at 1 and 10 μM cutoff with different fingerprints respectively.

From Tables 3 and 4 we observe that RWR performance is better for 1 μM target than 10 μM because at 10 μM we have lots off targets from different classes and as a result of that the prediction rate falls. For ChEMBL 1 μM dataset, drugs having more than 0, 1 and 2 targets we achieve BEDROC score of 0.433, 0.553 and 0.611, respectively, which is much better than a random set of interactions. To test whether random walk performs better than just a simple sequence similarity search we took the approved drugs and it’s known targets from the ChEMBL 10 μM dataset and performed sequence similarity based sech against 3,519 targets and ranked them. We found RWR performance is way better in ranking targets than performing simple sequence based search. The results are shown on Tables 3 and 4. This is the first time that the random walk-based method is evaluated using a binding assay dataset (cf. [3, 5]).

**Table 3:** Shows the types of data we used the drug target interaction having more than 1 and 2 drug interactions, area under the accumulated curve (AUAC), area under the ROC curve (AUC), bedroc and, enrichment factor (EF) and AUC Top 10%

Data types	Number of targets	AUAC (%)	AUC (%)	BEDROC	EF	AUC (Top 10%)
ChEMBL 1 μM (RWR)	>0	0.709	0.995	0.433	5.058	0.455
ChEMBL 1 μM (Seq)	>0	0.670	0.670	0.396	4.48	0.414
ChEMBL 1 μM (random RWR)	>0	0.494	0.493	0.075	1.090	0.079
ChEMBL 10 μM (RWR)	>0	0.596	0.837	0.323	3.865	0.351
ChEMBL 10 μM (Seq)	>0	0.518	0.518	0.237	2.641	0.2555
ChEMBL 10 μM (random RWR)	>0	0.394	0.364	0.036	0.954	0.029
ChEMBL 1 μM (RWR)	>1	0.784	0.784	0.553	6.286	0.569
ChEMBL 1 μM (Seq)	>1	0.652	0.651	0.390	4.507	0.412
ChEMBL 1 μM (random RWR)	>1	0.483	0.483	0.081	1.290	0.083
ChEMBL 10 μM (RWR)	>1	0.613	0.61	0.353	4.091	0.378
ChEMBL 10 μM (Seq)	>1	0.551	0.552	0.279	3.084	0.300
ChEMBL 10 μM (random RWR)	>1	0.514	0.514	0.075	1.244	0.088
ChEMBL 1 μM (RWR)	>2	0.823	0.824	0.611	6.866	0.631
ChEMBL 1 μM (Seq)	>2	0.701	0.705	0.513	5.109	0.469
ChEMBL 1 μM	>2	0.533	0.533	0.0671	1.465	0.065
ChEMBL 10 μM (RWR)	>2	0.632	0.633	0.399	4.569	0.422
ChEMBL 10 μM (Seq)	>2	0.569	0.569	0.298	3.03	0.315
ChEMBL 10 μM (random RWR)	>2	0.521	0.521	0.262	1.95	0.125

**Table 4:** Shows the types of data we used the drug target interaction having more than 1 and 2 drug interactions and sensitivity (hit rate) at top 10, 25, 50, 100 and 200 predicted targets

Data types	Number of targets	Top 10 (%)	Top 25 (%)	Top 50 (%)	Top 100 (%)	Top 200 (%)
ChEMBL 1 μM (RWR)	>0	0.144	0.342	0.470	0.532	0.607
ChEMBL 1 μM (Seq)	>0	0.164	0.315	0.394	0.420	0.430
ChEMBL 1 μM (random RWR)	>0	0.002	0.013	0.018	0.036	0.021
ChEMBL 10 μM (RWR)	>0	0.11	0.247	0.324	0.386	0.409
ChEMBL 10 μM (Seq)	>0	0.122	0.183	0.234	0.249	0.254
ChEMBL 10 μM (random RWR)	>0	0.014	0.023	0.035	0.048	0.079
ChEMBL 1 μM (RWR)	>1	0.274	0.477	0.550	0.580	0.614
ChEMBL 1 μM (seq)	>1	0.189	0.350	0.428	0.472	0.513
ChEMBL 1 μM (random RWR)	>1	0.007	0.023	0.038	0.076	0.091
ChEMBL 10 μM (RWR)	>1	0.220	0.277	0.348	0.417	0.446
ChEMBL 10 μM (seq)	>1	0.13	0.212	0.276	0.296	0.302
ChEMBL 10 μM (Random RWR)	>1	0.014	0.023	0.035	0.048	0.079
ChEMBL 1 μM (RWR)	>2	0.271	0.518	0.598	0.634	0.677
ChEMBL 1 μM (seq)	>2	0.19	0.393	0.53	0.56	0.598
ChEMBL 1 μM	>2	0.006	0.018	0.034	0.055	0.08
ChEMBL 10 μM (RWR)	>2	0.233	0.297	0.353	0.4299	0.472
ChEMBL 10 μM (seq)	>2	0.13	0.22	0.295	0.316	0.324
ChEMBL 10 μM (Random RWR)	>2	0.012	0.028	0.040	0.057	0.093

### Case study: profiling top selling drugs

**Fig. 5:**
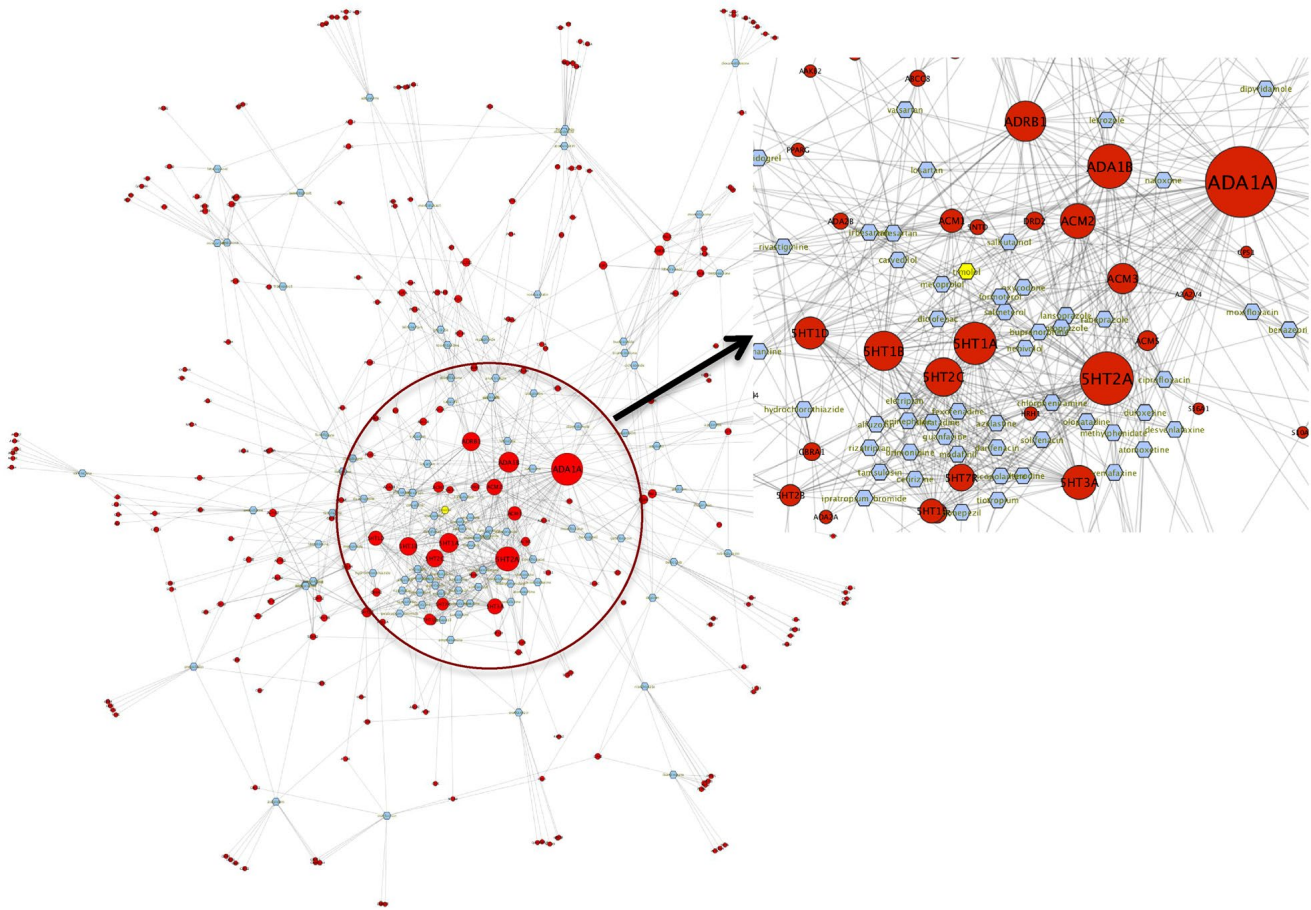
Shows the network of the top 10 predicted targets of 110 drugs. *Blue nodes* represent drugs and *red ones* represent targets. The size and label of the target nodes is proportional to the degree of the nodes.

Here, as a case study we investigate the target profiles of the popular top selling drugs in 2012 [18]. First, we consider $u_{\infty } ,$ the steady-state probability vector for the targets in our framework, as ‘target profile’ of a drug. Then we examine the top 10 predicted targets for the top selling drugs. We find that some targets are associated with many drugs (see Table 5). For instance, *adrenoceptor alpha 1A* appears in 60% of drug’s top 10 target association lists; *serotonin receptor 5HT2A* appear in 43%; and *adrenoceptor alpha 1B* in 35%. Most drugs shown on the Table 5 mostly belong to the rhodopsin class of GPCR’s. In Additional file 4, predictions are provided for 110 drugs with 3,519 targets and Fig. 5 shows a bipartite network of 110 drugs with top 10 predicted targets for each drug.

**Table 5:** The top 10 associated targets of 110 drugs with true percentage of associated target before prediction and predicted percentage of association

Targets	Percentage of drugs associated with the targets based on DrugBank and ChEMBL (%)	Percentage of drug associations appearing in top-10 target list in our prediction (%)
*ADA1A*	7.27	60
*5HT2A*	4.54	43.63
*ADA1B*	7.27	35.45
*5HT1A*	4.54	33.63
*ADRB1*	5.45	31.81
*5HT1B*	5.45	30.90
*5HT2C*	3.63	30
*ACM2*	9.09	26.36
*5HT3A*	4.54	25.45
*5HT1D*	5.45	23.63
*ACM3*	9.09	21.81
*5HT7R*	4.54	18.18

We took some random drugs and tried to find known binding associations to protein targets. We searched three databases ChEMBL [19], PDSP [20], and Pubchem [21] using the binding coefficients like IC50 and Ki. Table 6 lists the 10 predicted drug–target associations that we have identified evidence of binding interaction in other databases. These findings suggest that these targets may have many undiscovered interactions with existing drugs. Further investigation may have significant values on understanding side effects of existing drugs as well as repurposing them.

**Table 6:** Drug target interactions with association values from different databases

Protein	Drug	Source	Activity Tpe	Activity μM
*5HT2A*	Carvedilol	Pubchem AID 625192	IC50	0.41
*5HT2A*	Desloratadine	Pubchem AID 625192	IC50	0.033
*KCNH2*	Lidocaine	ChEMBL	IC50	263.02
*ADRB1*	Salmetorol	ChEMBL	IC50	0.501
*5HT1A*	Amphetamine	PDSP database	Ki	6.6
*HDAC2*	Atorvastatin	ChEMBL	IC50	22.5
*ADA1A*	Duloxentine	PDSP	Ki	10
*ACM1*	Montelukast	Pubchem AID 625153	IC50	8.045
*SC6A4*	Quetiapine	PDSP	Ki	10

Finally, let us summarize the contributions of this paper. First, we offer a general approach that takes the whole drug target network into account without separating protein categories, in contrast to the previous study [3]. The following estimation corroborates our approach. Our drug-target dataset contains 727 drugs and 3,519 proteins. The number of interactions between drugs and targets is 2,557, which makes 684 drugs to have at least one known target and 457 drugs to have two or more interactions. The proteins in the dataset are grouped under 15 different categories according to ChEMBL target classifications (https://www.ebi.ac.uk/chembl/target/browser). Out of 3,519 proteins, 1,386 proteins belong to one of the categories and other proteins do not have category information. The number of drugs that have at least two interactions with proteins that are categorized is 412. Among these 412 drugs, the number of drugs that have interactions with proteins from multiple groups is 169. In other words, we estimate that about 40% of drugs have interactions across multiple groups according ChEMBL dataset. Therefore, it is more reasonable to consider all proteins together, rather than running the prediction model separately for each category.

Second, we further investigate the methodology by presenting a benchmark of a parameter ƞ in conjunction with the four chemical fingerprint types: MACCS 166 keys, ECFP6 fingerprints, PHFP4 fingerprints, and ROCS. In the previous study, the parameter space of ƞ is not explored below 0.1, but we find that we can improve the performance by decreasing eta below 0.1. We also find that the performance is robust under the choice of chemical fingerprinting method, particularly when ƞ is around the optimum (~0.01). Very small $\eta$ eta means the walk in the target network is much more important than the walk on the drug–drug network. In a sense, it indicates that drug network add some information but only marginally. And also the drug network is not very useful in prioritizing targets.

## Conclusion

We have demonstrated that RWR approach provides a powerful way of predicting of drug–target interactions. There are two significant benefits of the approach. First, it provides a natural way to integrate multiple types of information such as drug–drug similarity, target–target similarity, and existing drug–target interactions into a coherent framework. Second, in contrast to other approaches like short-path-based methods, the random walk framework incorporates the network structure around a single or multiple points of interests extensively, taking into account not only the closeness of targets, but also the multitude of the paths to the targets. These properties allow us to predict novel targets even for the drugs that have no known target, by connecting such drugs to the network through the drug–drug similarity. Still, the performance of RWR could be further improved by incorporating more known drug–target interactions. We have studied the performance of the method under the variations of ƞ parameter and the choice of fingerprints methods, showing that while training the model one can use any of the chemical features as similarity matrix with parameter $\eta = 0.0 1$ to obtain the predicted results, without significantly affecting the outcomes.

## Additional files

The following are the datasets used for these experiments.
Additional file 1: Binary Drug Target matrix consisting of 727 drugs and 3,519 proteins. Additional file 2: Four Drug–Drug similarity matrices. Additional file 3: Predictions Results. Additional file 4: 110 Drugs predicted results with 3,519 targets.
